# Occupational rehabilitation programs for musculoskeletal pain and common mental health disorders: study protocol of a randomized controlled trial

**DOI:** 10.1186/1471-2458-14-368

**Published:** 2014-04-16

**Authors:** Marius S Fimland, Ottar Vasseljen, Sigmund Gismervik, Marit By Rise, Vidar Halsteinli, Henrik B Jacobsen, Petter C Borchgrevink, Hanne Tenggren, Roar Johnsen

**Affiliations:** 1 Department of Public Health and General Practice, Faculty of Medicine, Norwegian University of Science and Technology, Trondheim, Norway; 2 Hysnes Rehabilitation Center, St. Olavs University Hospital, Trondheim, Norway; 3 Department of Physical Medicine and Rehabilitation, St. Olavs University Hospital, Trondheim, Norway; 4 Department of Circulation and Medical Imaging, Faculty of Medicine, Norwegian University of Science and Technology, Trondheim, Norway

**Keywords:** Absenteeism, Occupational health, Rehabilitation, Return to work, Cognitive behavior therapy, Exercise musculoskeletal diseases

## Abstract

**Background:**

Long-term sick leave has considerably negative impact on the individual and society. Hence, the need to identify effective occupational rehabilitation programs is pressing. In Norway, group based occupational rehabilitation programs merging patients with different diagnoses have existed for many years, but no rigorous evaluation has been performed. The described randomized controlled trial aims primarily to compare two structured multicomponent inpatient rehabilitation programs, differing in length and content, with a comparative cognitive intervention. Secondarily the two inpatient programs will be compared with each other, and with a usual care reference group.

**Methods/design:**

The study is designed as a randomized controlled trial with parallel groups. The Social Security Office performs monthly extractions of sick listed individuals aged 18–60 years, on sick leave 2–12 months, with sick leave status 50% - 100% due to musculoskeletal, mental or unspecific disorders. Sick-listed persons are randomized twice: 1) to receive one of two invitations to participate in the study or not receive an invitation, where the latter “untouched” control group will be monitored for future sick leave in the National Social Security Register, and 2) after inclusion, to a Long or Short inpatient multicomponent rehabilitation program (depending on which invitation was sent) or an outpatient cognitive behavioral therapy group comparative program. The Long program consists of 3 ½ weeks with full rehabilitation days. The Short program consists of 4 + 4 full days, separated by two weeks, in which a workplace visit will be performed if desirable. Three areas of rehabilitation are targeted: mental training, physical training and work-related problem solving. The primary outcome is number of sick leave days. Secondary outcomes include time until full sustainable return to work, health related quality of life, health related behavior, functional status, somatic and mental health, and perceptions of work. In addition, health economic evaluation will be performed, and the implementation of the interventions, expectations and experiences of users and service providers will be investigated with different qualitative methods.

**Trial registration:**

ClinicalTrials.gov: NCT01926574.

## Background

Long-term sickness absence has considerable impact on social functioning, on the families of the sick-listed, the companies they work for, and society as a whole [1]. Therefore, measures to reduce likelihood of dropping out from work have been implemented through legislation, Health, Safety and Environment acts and by different treatment and rehabilitation services. Throughout many European countries various outpatient and inpatient rehabilitation programmes have been established to prevent long-term sickness absence and permanent work disability. However, the documented effects of work rehabilitation on working capacity, work participation and health are sparse [2].

Most work rehabilitation programs described in the scientific literature are designed for single or a specific diagnostic group. Several studies have investigated return-to-work (RTW) interventions for musculoskeletal disorders [3], particularly for workers with non-specific low back pain [4-7]. Far fewer studies are conducted for workers with mental problems, although RTW programs have been investigated in absenteeism due to mental disorders [8], distress [9], adjustment disorders [10] and depression [11]. It is now recognized that many patients on sick leave have more than one health complaint. As an example, previous studies of workers on sick leave due to chronic low back pain in Norway showed that less than 2% reported low back pain as their only complaint [12]. In addition, a recent study on sick listed patients with low back pain showed that one out of three had psychiatric comorbidity [13]. Similar findings are reported from other western countries [14,15], demonstrating a substantial overlap of musculoskeletal, mental, and other disorders.

The overlap and complexity of health complaints makes it difficult for general practitioners to agree on the diagnosis of patients with several health complaints, and the diagnosis given to patients with similar symptoms may vary greatly between general practitioners [16]. Since there is considerable degree of comorbidity among these patients, the present trial will employ a comprehensive rehabilitation model suited for patients with musculoskeletal disorders as well as common mental disorders and/or unspecific disorders. This is in line with the paradigm shift of occupational medicine from disease treatment to disability rehabilitation and management [17,18].

In Norway, tertiary institutional care occupational rehabilitation programs have been active for more than 27 years. Although some evaluations have been performed [19,20] none have used a randomized research design. Thus, the effects on work participation and health outcomes of institutional occupational rehabilitation programs are largely unknown. Further, institutional occupational rehabilitation programs in Norway usually last ~ four weeks, and the patients stay at the center during this period. However, the rationale for a four-week rehabilitation period with full rehabilitation days is based on experience and convenience rather than scientific evidence. Hence, rehabilitation programs with different durations should be investigated. In addition, physical activity/exercise and coping of health complaints are emphasized in several of these programs, whereas there is generally little work place involvement [21]. Conversely, RTW-programs described in the scientific literature have suggested that structured meetings between employee, employer and occupational health professionals are important for improving RTW rates [22,23].

The primary purpose of this study is to explore the effect of two different multicomponent work-rehabilitation programs on sickness absence, motivation for work, somatic- and mental health, and related outcomes. The *Long* program consists of 3 ½ weeks with full rehabilitation “work” days. The *Short* program consists of 4 + 4 full days of rehabilitation, separated by two weeks. In the Short program a workplace visit will be performed, in addition to the 4 + 4 rehabilitation days, if considered relevant by the worker and the rehabilitation team. A single-component psychological intervention will serve as a comparative control arm. Using a design where participants will be randomized twice, both the Long and Short program will be compared with the comparative control arm in separate randomized controlled studies, but in addition we will also compare the Long with the Short program. Furthermore, as any form of contact can be perceived as an intervention, an ‘untouched’ control group will also be followed in national registers and used as a usual care reference.

### Objectives

**Figure 1:**
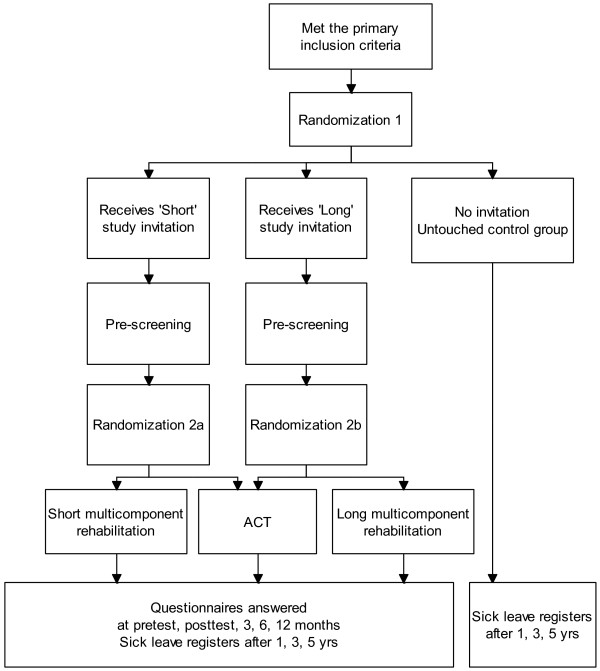
**Design of the study.** ACT: Comparative outpatient Acceptance and Commitment Therapy Rehabilitation group.

In line with current recommendations [24,25], the objective of this protocol article is to describe the design of a randomized controlled trial, including health economics evaluation and qualitative implementation studies alongside the trial. The design with randomization at two levels makes several comparisons possible (Figure 1). This study will investigate the effects of short- and long-term inpatient multicomponent rehabilitation programs compared to a single-component intervention and an ‘untouched’ usual care reference group from sick-leave registers. The latter will serve as a reference group to the three interventional groups. The principal objectives are to explore the following research questions:

• Are the multicomponent rehabilitation programs more effective in reducing sickness absence compared to the single component Acceptance and Commitment Therapy (ACT) program (i.e. Short vs. ACT; Long vs. ACT), and are there differences between the Long and Short programs?

• Are the multicomponent rehabilitation programs cost effective compared to the single component ACT program (i.e. Short vs. ACT; Long vs. ACT)?

• Are the patients’ and service providers’ expectations before participation in the multicomponent rehabilitation programs, in accordance with their experiences afterwards?

• According to the patients’ and service providers’ experiences; have the multicomponent rehabilitation program been implemented as planned?

• Are the multicomponent programs more effective in improving secondary outcomes (e.g. mental or physical health, or motivation for return to work) than the single component Acceptance and Commitment Therapy (ACT) program?

## Methods/design

### Project context

This research project was initiated to evaluate a new occupational rehabilitation centre, Hysnes Rehabilitation Centre (http://www.stolav.no/hysneshelsefort), established as part of St. Olav’s University Hospital. The centre is located in the municipality of Rissa, a 50 min boat ride or 90 min drive from Trondheim, the third largest city (181.500 inhabitants) in Norway. The two multicomponent interventions to be investigated are provided at the rehabilitation centre and consist of a long and a short inpatient program. The control arm, the single component out-patient intervention (see below), is provided at St. Olav’s University Hospital in Trondheim. Earmarked funding from the national budget was provided over a five-year period (2010–2014) to establish and run the rehabilitation programs, inclusive funding for scientific evaluation.

### Design

The study is designed as a randomized controlled trial with three parallel groups and will be reported according to the CONSORT statement [26]. The study includes health economic- and qualitative aspects. The design is presented in Figure 1. Monthly extractions of sick listed individuals from The Norwegian Labour and Welfare Service (NAV) with the targeted diagnoses serving as the recruitment base for the study population.

### Study population

Persons living in the counties of Trøndelag, population of 375 000 inhabitants, are eligible for inclusion if they are 18–60 years old and have been on sick leave 2–12 months, with a current sick leave status of 50% - 100%. Individuals with an ICPC-2 (International Classification of Primary Care, Second edition) diagnosis within the L (musculoskeletal), P (psychiatric) or A (unspecific disorders) categories are eligible. Thus both persons being on sick leave due to musculoskeletal and psychological disorders, chronic pain, fatigue and other non-specific disorders are included in the study. Exclusion criteria are: 1) alcohol or drug abuse; 2) serious somatic (e.g. cancer, heart disease) or psychological disorder (e.g. suicide attempts, psychosis, ongoing manic episode); 3) a specific disorder requiring specialized treatment; 4) pregnancy; 5) currently participating in another treatment program; 6) insufficient comprehension of Norwegian language to participate in group sessions and to fill out questionnaires; 7) scheduled for surgery within the next 6 months; and 8) serious problems with functioning in a group setting.

### Recruitment procedure

NAV provide monthly lists of persons meeting the inclusion criteria. The assigned project co-worker checks eligibility performs the first randomization (Figure 1) and sends out invitations to potential participants. They receive available information of the project through written leaflets, website of the rehabilitation centre and are invited to call a project co-worker if they have queries. The potential participants are asked to answer a two-page questionnaire and a consent form indicating whether they agree or disagree to take part in the study, or if they require more information. Contacted persons are asked to reply to the project co-worker using an enclosed prepaid envelope. Positive answers are forwarded to the researchers who determine initial eligibility based on the short questionnaire. Prior to enrolment in the study a physician, psychologist and physiotherapist will perform an outpatient assessment of eligibility. Results from the outpatient assessment will be provided to the relevant health personnel and utilized in the rehabilitation program.

### The three occupational rehabilitation interventions

The three intervention programs: Long and Short multicomponent in-patient programs and the single component program are developed through cooperation between health care personnel and the research group and have all been piloted. All health personnel delivering the interventions were given training in ACT prior to and during (monthly) the intervention by the same certified ACT-supervisor. See Table 1 for an overview and comparison of the programs.

**Table 1:** Overview of the three rehabilitation programs

	**Long multicomponent program**	**Short multicomponent program**	**ACT comparative arm**
*Setting*	Inpatient rehabilitation center	Inpatient rehabilitation center	Outpatient Hospital clinic
*Duration*	3.5 weeks	4 + 4 days, separated by 2 weeks living at home	6 weeks
*Contents and quantities*	-group discussions (×8, total 16 h; ACT based)	-group discussions (×6, total 12 h; ACT based)	-ACT group discussions (×6, total 15 h)
	-psychoeducational sessions (×4, total 6.5 h)	-psychoeducational session on stress (×1, 2 h)	-group discussion on physical activity (×1, 1 h)
	-individual meetings with coordinator (×5, total 5 h)	-individual meetings with coordinator (×2, total 2 h)	-individual sessions with social worker (×2, total 2 h)
	-individual meeting with physician (×1, 0.5 h)	-individual meeting with physician (×1, 0.5 h)	-individual session with social worker and ACT group moderator (×1, 0.5 h)
	-mindfulness sessions (×7, total 3.5 h)	-mindfulness sessions (×4, total 2 h)	-home practice, including daily mindfulness
	-individual/group based supervised training sessions (×10, total 12 h)	-individual/group based supervised training sessions (×8, total 10.5 h)	
	-“walking to work” (×6, total 3 h)	-Create RTW-plan	
	-Create RTW-plan	In the 2 weeks between the stays at the rehab:	
	-outdoor activities day (×1, 5 h)	-Meeting with employer, if relevant and permitted	
	-“network day” with 2 group sessions (total 4 h)	-At least 2 contacts with team coordinator (telephone or personal)	

All references to time are average estimates, which may include short breaks in and between sessions. ACT: Acceptance and Commitment Therapy. RTW: Return-to-work.

*The Long and Short multicomponent programs* are both individual and group-based with maximum eight participants in each group organized as a six-seven-hour workday at the inpatient rehabilitation center. The Long program lasts ~3.5 work weeks (17 days) and the Short program lasts 4 + 4 days separated by 2 weeks where participants live at home. In both programs, 2–3 designated RTW coordinators per group are involved in coordinating and executing the interventions. Three areas of rehabilitation are targeted: mental training, physical training and work-related problem solving. The coordinator’s backgrounds are diverse (physical therapy, psychology, and exercise physiology, nursing or other). Each coordinator is responsible for mentoring two or three participants during the intervention. There are three multidisciplinary team meetings, where both health personnel in the Long and Short programs attend, discussing strategies concerning the participants’ obstacles and possibilities for increased work participation (total 5, 5 hours).

*The mental training*, aims to increase the participant’s psychological flexibility, motivation and self-efficacy, and reduce sickness absence. The intervention manuals are based on ACT (Acceptance and Commitment Therapy) [27], an evidence-based cognitive-behavioral approach. The therapeutic model in ACT is founded around six dynamic processes: committed action, self-as-context, presence in the moment, values, defusion and acceptance. These processes are targeted both in group-sessions and individual meetings. To ensure that all six processes are addressed in the group-sessions, the coordinators evaluate which of the processes they have targeted after each session. Commitment, value-based actions, and being mindful are intended to increase motivation and efficacy with regard to work. The mental training consists of group sessions, psycho-education (stress, pain and symptom interpretation, nutrition, and sleep in the Long program, and only on stress in the Short program). Further, mindfulness training and individual homework are given in all programs.

*The physical training* intervention aims to increase physical strength and endurance capacity and to promote physical activity, whilst also improving knowledge about physical activity. It also targets fear of movement, tension-related pain, and a “normal” pattern of movement. Each participant starts their stay developing a personalized physical activity plan in cooperation with their coordinator and the designated exercise coach. The teaching and training sessions are both individual and group-based. The exercise program is individualized based on clinical judgment, and the goals and wishes of the participant. The exercise coach regularly evaluates the participant’s progress. Participants are given training programs when leaving the center.

*The work-related problem-solving* aims to identify challenges and possibilities, increase readiness for work and ways to adopt these strategies in daily life after the stay at Hysnes rehabilitation center. This is performed as part of the group sessions and in the individual meetings with the coordinator. This intervention aims to motivate the participant, clarify the value of work, to highlight participants’ challenges and resources, and to make a realistic plan for increasing their work participation. This plan is being worked on throughout the rehabilitation stay. Moreover, the crossover from Hysnes Rehabilitation Center to re-enter work and home life is addressed. Stakeholders in the rehabilitation process are included in the process: midway and discharge summaries are sent to the participant’s general practitioner in all cases, and to the social security office and employer, depending on relevance and the participants’ consent. Furthermore, there are different stakeholder-involvement in the Short and Long program, as described below.

*Differences between the Long and Short programs*. In addition to differences in duration and amount, there are some content differences between the Long and Short multicomponent programs. In the Long program a “network day” is arranged where the participant can bring one or several persons (usually family, physician or close friends) to gain insight in the rehabilitation process, in order to facilitate support after the end of the program. In the Short program, a workplace visit is arranged if relevant and supported by the participant, either between the two rehabilitation stays or after the stay at Hysnes rehabilitation center. The purpose of this visit is to identify barriers and solutions for RTW. If the participant currently has no job, a meeting with The Social Security Office (NAV) may be conducted instead. The general practitioner can also attend if relevant. Meetings are usually performed in person, but can also be arranged via video or telephone conference. While participants in the Short program are at home, the coordinator and participant have at least two contact points.

*The single component ACT comparison intervention* is provided in the context of an established musculoskeletal outpatient specialist unit at the Physical Medicine and Rehabilitation (PM&R) department at St. Olav’s University Hospital. This program is an outpatient group intervention using ACT in groups following a manual especially designed for this study.

A maximum of nine participants are invited to attend group-sessions once a week for six weeks. One of two physicians (both PM&R specialists) or a psychologist all specifically trained in ACT is in charge of the group sessions. Home practice is encouraged between sessions including 15 min daily mindfulness practice. In addition, an experienced social worker with training in occupational rehabilitation and ACT offers two individual sessions. The individual sessions aim to clarify personal values and problem solving issues if considered relevant for increased work participation. Participants are also invited to take part in a physiotherapist lead group discussion on benefits and motivational factors for physical exercise. Physical exercise is not included in the intervention.

The intervention ends with an individual session with both the social worker and the ACT group moderator present. In this session the participant contributes to and approves a final summary letter addressed to the general practitioner. The letter summarizes the intervention content, the participant’s experiences during the six weeks and future plans of action. No further steps are made for coordination between stakeholders. Participants are encouraged not to start any new treatment during the intervention but are allowed to continue any concurrent treatment at the discretion of the general practitioner.

### Outcomes

#### Primary outcome

Total number of sickness absence days during the 6 months after enrolment in the study (i.e. after pre-screening), obtained by national registers.

#### Secondary outcomes and additional measures

Secondary outcomes include additional sickness absence measurements, health related quality of life, health related behavior, functional status, somatic- and mental health, and perceptions of work and returning to work. Particularly we will measure:

• Time until full sustainable RTW (i.e. for at least 4 weeks without relapse).

• The proportion of workers at work will be obtained by national registers and a self-report of social security benefits received from NAV (The Social Security Office).

• One, three and five-year follow-up of total registered days of physician referred sick leave by national registers.

• Readiness, beliefs and motivation for Return to Work, measured by Readiness for Return to Work Scale [28] and the questions: “How long do you think you will be on sick leave from today” (Not at all, less than 1 month, 1–2 months, 2–4 months, 4–10 months, more than 10 months), “Do you want to return to work” (yes/no), “How strongly do you want to return to work” (not at all-very much, 1, 2…-10).

• Health-related quality of life by 15D (15 dimensions) [29].

• Perceived general health with the question: How is your health now?

• Pain intensity and pain sites by a body pain chart; and question 3–5 from the Brief Pain Inventory [30].

• Catastrophizing thoughts regarding pain by two questions from the Coping Strategies Questionnaire [31].

• Symptoms of depression and anxiety by the Hospital Anxiety and Depression Scale [32].

• Subjective health complaints by the SHC Inventory [33].

• Physical activity levels measured by the International Physical Activity Questionnaire [34], and three additional items concerning frequency, intensity and duration of exercise from the third wave of the HUNT study [35].

• Physical, social and emotional functional status measured by the first four charts of COOP/WONKA [36].

• Four subscales (Job Demands, Control at work, Mastery of work and Social interactions at work) from The general Nordic questionnaire for psychological and social factors at work (QPSnordic) [37].

• Duration and intensity of complaints and interruption with work tasks. The original questions referred to pain only [38], but we modified them so they would apply to all complaints.

• Fear of movement in relation to work and physical activity, with the Fear Avoidance Beliefs Questionnaire [39].

• Psychological flexibility and acceptance by the Acceptance and Action Questionnaire-II [40].

#### Health economics

Cost-effectiveness and cost-utility will be evaluated from a societal perspective where both direct and indirect costs will be measured according to Norwegian guidelines for economic evaluation [41]. Direct costs comprise health services used, while indirect costs are loss of productivity due to sick leave. Treatment costs will be estimated specifically for the two interventions and for the comparative intervention based on standardized individual patient programs and a micro-costing approach. In the cost-effectiveness analysis, sickness absence days will be used as outcome measure, and to avoid double counting productivity costs will be excluded. In the cost-utility analysis, the outcome measure is Quality Adjusted Life Years based on the 15D instrument (22), and productivity costs will be included. In both cost-effectiveness and cost-utility analysis, the incremental ratio will be calculated by dividing the incremental cost by the incremental effect. Bootstrapping procedures will be used to estimate uncertainty surrounding the cost-differences and the incremental ratio. Sensitivity analysis will be applied and cost-effectiveness plane and acceptability curves used for additional presentation purposes.

#### Qualitative studies

Some subjects will also be asked to participate in a qualitative study linked to the occupational rehabilitation intervention. The primary purpose of the qualitative studies is to reveal the participants’ and providers’ perception and experience with implementation of the rehabilitation programs. The study will further focus on the participants’ expectations before taking part in a multicomponent rehabilitation program and their experiences afterwards. In addition, the service providers’ rationale and experiences with the rehabilitation programs will be explored and compared to the participants’ experiences. The study will also explore facilitators and barriers to the RTW process as perceived by the different stakeholders (participants and providers). Data will be analysed according to the method of grounded theory [42].

### Data collection

Data on sickness benefits and other social benefits will be based on register data from Statistics Norway as long as 5 years after participation in one of the rehabilitation programs. Self-reported data will be collected by electronic questionnaires via the internet (http://www.checkware.com) before pre-screening, at pre-test, post-test, and 3, 6 and 12 months after pre-test. Qualitative data will be collected through semi-structured focus group interviews, through individual interviews, and participant observations. Interviews will be based on an interview guide, audio recorded and transcribed. Some participants will keep notebooks during and after the rehabilitation program to explore their experience of the RTW process.

### Sample size

Three approaches have been performed to determine sample size:

1) Comparison of number of days with sick leave at 6 months of follow-up (P = 0.05; 90% power): An average of 60 days (SD 40) and 90 days (SD 60) of sick leave in the intervention and comparative group, respectively would require 61 persons for each group.

2) Comparison of time to sustainable RTW with Kaplan Meier survival analysis with log rank test with a hazard ratio of 0.6 (alpha 0.05, beta 0.20) would require 63 in each group (secondary outcome).Comparison of the share of workers after one year with the same statistical assumptions as point 1; 60% versus 40% RTW would require 68 people in each group. (secondary outcome).

Accordingly, with estimated ~20% loss to follow-up we will include 80 persons in each arm.

### Randomization

Sick-listed persons will be randomized twice (see Figure 1). Firstly, subjects sick listed in the Social Security System will randomly receive one of two invitations (Long or Short program study) to participate in the study. Those allocated to not receive an invitation will serve as an unaware control group, and their future sick leave monitored. Secondly, invited subjects who provide informed consent and are found eligible, will be randomized to the control arm (single-component ACT-program) or to the already allocated Long or Short multicomponent rehabilitation program (depending on the outcome of randomization 1). Hence there are two separate randomized controlled studies at this level (Long vs. ACT and Short vs. ACT). Both randomizations are blinded. The first randomization will be performed by a project co-worker; and the second randomization by the Unit of Applied Clinical Research (third-party) at the Norwegian University of Science and Technology (NTNU). With anonymous aggregated data, we can also compare the reference group with those who 1) do not reply to the invitation, 2) decline participation and 3) are excluded after the pre-examination.

### Ethical considerations

REC Central - the Regional Committee for Medical and Health Research Ethics in Central Norway has approved these studies (No.: 2012/1241), and the trial is registered in clinicaltrials.gov (No.: NCT01926574). All participants enrolled in the study will take part in one of three rehabilitation programs in the specialist health care at St. Olav’s University Hospital. Those randomized to not being invited receive usual care, and the researchers will only be given anonymous aggregated information on outcome, sick-leave and sustainable RTW.

### Statistical analyses

Effect analyses of primary and secondary outcomes will be performed according to the intention to treat principle, and per protocol. For the primary outcome, difference in days of sick leave will be evaluated with the Mann Whitney U-test, as sick leave days are unlikely to be normally distributed. Sustainable RTW will be evaluated with survival analysis. Kaplan-Meier analysis will be used to describe association between groups and the duration of absence from work until sustainable RTW. Mixed models will be used to analyze time-dependent health outcomes and to estimate between group differences over time.

## Discussion

Sick leave is a major problem in the western world. Norway is considered to have a high level of sick leave [43], and approximately 10% of the workforce is on disability pension [44]. Hence, it is necessary to develop services that help people to stay at work, including effective rehabilitation models.

### Strengths

This trial will be the first to investigate whether a group based rehabilitation program for patients with musculoskeletal, mental or unspecific problems can facilitate work participation, whereas comparable studies have employed rehabilitation programs tailored for populations with a specific disorder (e.g. low back pain). Employing a national register for sickness absence data eliminates recall bias and provides data from non-responders. Furthermore, our design with randomization at two levels will make it possible to assess the effects of all interventions, and also determine the effect of contacting potential participants. Sending an invitation letter instead of recruiting from e.g. general practitioners will ensure that the group is not biased by referral, improving generalizability of the results. Moreover, the RCT will be followed by economic evaluations. Finally, the qualitative studies aim to explore the implementation of the rehabilitation programs more in depth. They will shed light on barriers and facilitators for implementation of such programs by studying the participants’ and providers’ perceptions before taking part in the intervention. A realistic evaluation approach would help to describe what providers and participants perceive as the important, helpful, or difficult aspects of an inpatient multicomponent rehabilitation program.

### Limitations

There are some limitations with this study. As in all interventions of this nature, blinding of participants or care providers is not possible. However, regardless of randomization outcome, all participants are given high quality rehabilitation by health professionals at St. Olav’s University Hospital. As in other countries, Norway has its distinct socio-economic and socio-cultural context. Therefore, the results cannot necessarily be generalized to other contexts. This occupational rehabilitation program is specifically tailored the Norwegian sickness benefit-, work-, health-, political systems and culture. Hence, implementation of these programs in other settings should be preceded by necessary modifications of the program.

### Impact of results

The knowledge to be generated will be important for both policy makers and clinicians and other professionals in practice. Decision makers will be given the best possible information, concerning the effect of this type of rehabilitation program. Furthermore, they will guide existing and new occupational rehabilitation programs. The main results of these studies will be published in 2015/16.

## Competing interests

The authors declare that they have no competing interest.

## Authors’ contributions

MSF was in charge of writing the article. MSF, OV, SG, VH and RJ contributed to the design of the RCT. VH is responsible for and wrote the section on health economics. MB is responsible for and wrote the section on qualitative studies. HT and PCB were responsible for the multicomponent programs. HBJ wrote the first draft of the description of the multicomponent programs. SG was responsible for the comparative intervention and wrote the description of this program. RJ and OV are the principal investigators of the project and initiated the study. All authors contributed to the writing of the article, and read and approved the manuscript.

## Pre-publication history

The pre-publication history for this paper can be accessed here:

http://www.biomedcentral.com/1471-2458/14/368/prepub
